# Risks and Function of Breast Cancer Susceptibility Alleles

**DOI:** 10.3390/cancers13163953

**Published:** 2021-08-05

**Authors:** Saeideh Torabi Dalivandan, Jasmine Plummer, Simon A. Gayther

**Affiliations:** Center for Bioinformatics and Functional Genomics, Department of Biomedical Sciences, Cedars Sinai Medical Center, Los Angeles, CA 90048, USA; saeideh.torabidalivandan@csmc.edu (S.T.D.); jasmine.plummer@cshs.org (J.P.)

**Keywords:** breast cancer risk, GWAS, subtype-specific risk, functional genomics, clinical genetic testing

## Abstract

**Simple Summary:**

Population-based genetic risk stratification and detection of early-stage breast cancers will improve approaches to prevent and reduce disease-associated mortalities. In this review, we summarize the latest discoveries in breast cancer susceptibility genetics and propose how these findings can be applied in the clinical arena to improve risk prediction and prevention of breast cancer. We also review the latest approaches and progress aimed at elucidating the functional consequences of both high and moderate penetrance genetic variation, which tend to lie in the protein coding regions of breast cancer susceptibility genes, and common low penetrance breast cancer risk alleles which tend to lie in non-protein coding DNA regions and affect gene regulation. For non-coding risk variation, there is no genetic code to interpret the function of common risk allele; and so, we provide the reader with an illustration of the step-by-step methods to understand their functional impact on breast cancer disease biology.

**Abstract:**

Family history remains one of the strongest risk factors for breast cancer. It is well established that women with a first-degree relative affected by breast cancer are twice as likely to develop the disease themselves. Twins studies indicate that this is most likely due to shared genetics rather than shared epidemiological/lifestyle risk factors. Linkage and targeted sequencing studies have shown that rare high- and moderate-penetrance germline variants in genes involved in the DNA damage response (DDR) including *BRCA1*, *BRCA2*, *PALB2*, *ATM*, and *TP53* are responsible for a proportion of breast cancer cases. However, breast cancer is a heterogeneous disease, and there is now strong evidence that different risk alleles can predispose to different subtypes of breast cancer. Here, we review the associations between the different genes and subtype-specificity of breast cancer based on the most comprehensive genetic studies published. Genome-wide association studies (GWAS) have also been used to identify an additional hereditary component of breast cancer, and have identified hundreds of common, low-penetrance susceptibility alleles. The combination of these low penetrance risk variants, summed as a polygenic risk score (PRS), can identify individuals across the spectrum of disease risk. However, there remains a substantial bottleneck between the discovery of GWAS-risk variants and their contribution to tumorigenesis mainly because the majority of these variants map to the non-protein coding genome. A range of functional genomic approaches are needed to identify the causal risk variants and target susceptibility genes and establish their underlying role in disease biology. We discuss how the application of these multidisciplinary approaches to understand genetic risk for breast cancer can be used to identify individuals in the population that may benefit from clinical interventions including screening for early detection and prevention, and treatment strategies to reduce breast cancer-related mortalities.

## 1. Introduction

Breast cancer is the most common cancer among women worldwide accounting for around 12% of all female cancers [1]. The American Cancer Society estimates that in the US in 2021 alone, there will be around 281,000 and 49,000 new female cases of invasive and non-invasive breast cancer, respectively, resulting in about 44,000 deaths. For non-invasive breast cancer, the overall 5- and 10-year survival rates are approximately 90% and 84%, respectively [2]. There has been an overall 41% decline in cancer death rate since 1989 although the decline in death rate has slowed over the past decade [2,3]. Approximately, 5–10% of breast cancers are hereditary, i.e., due to genetic predisposition [4]. The genes discovered so far that predispose to breast cancer account for about 25–30% of hereditary breast cancer [5], but a large proportion of patients with a family history of breast cancer do not carry risk variants in these genes, indicating that other genetic risk factors likely exist. Identifying the missing heritability will enable improved accuracy in genetic risk estimation in the population, identify novel susceptibility genes, increase our fundamental understanding of disease drivers, and enable approaches to translate these findings into clinical practice for early detection and prevention in individuals identified at risk of breast cancer. In this review, we discuss the different categories of genetic risks associated with breast cancer, with a focus on subtype-specific risks and review the functional approaches that are being used to interpret the biological role of risk variants and genes in breast cancer pathogenesis. Additionally, we discuss the clinical significance of genetic risk–based stratification of breast cancer in the population, at both prevention and treatment stages. In accordance with the latest professional guidelines, the term sequence variants or variants will be used throughout this article instead of mutation.

## 2. Genetic Predisposition to Breast Cancer

### 2.1. High- and Moderate-Penetrance Susceptibility Genes

**Figure 1:**
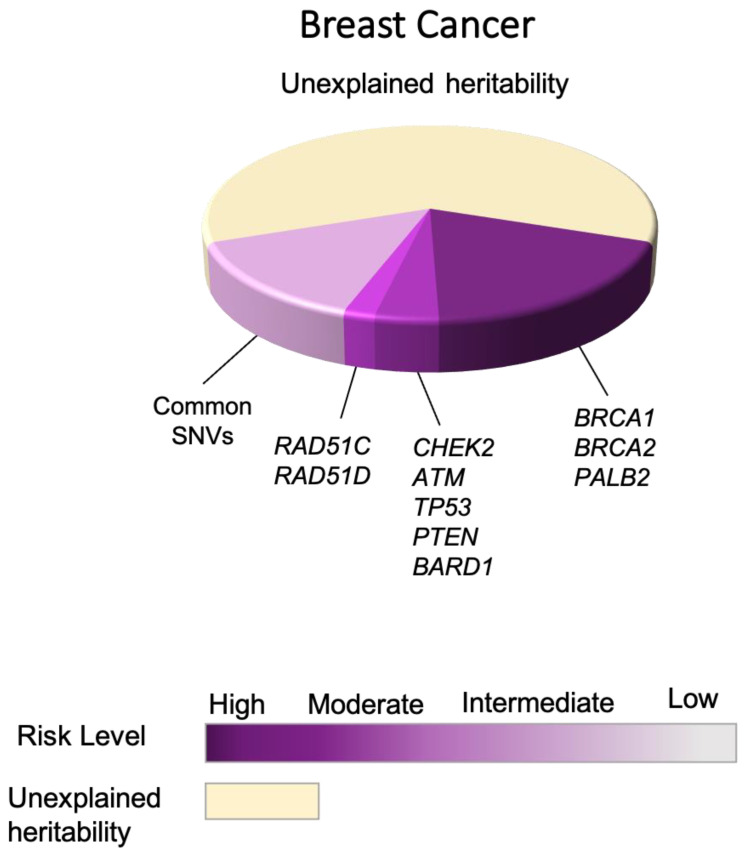
Illustration of the proportional contributions of breast cancer susceptibility risk genes (high- and moderate-penetrance genes), and common risk alleles to breast cancer.

The identification of the first breast cancer susceptibility genes dates back to the nineties when linkage studies led to the identification of the *BRCA1* and *BRCA2* genes (BReast-CAncer susceptibility gene 1 and 2) carrying likely pathogenic variants that co-segregate with breast cancer in families [6,7]. Female carriers of *BRCA1* or *BRCA2* pathogenic variants have an ~70% lifetime risk of being diagnosed with breast cancer by age 80 [8,9]. Male *BRCA1* and *BRCA2* carriers are also at an increased risk of breast cancer with risk estimates of ~1–5% and 5–10% respectively, compared with the general male population where the life-time risks are ~0.1% [10]. The BRCA1 and BRCA2 proteins are described as tumor suppressors [11] and have been found to play a significant role in DNA damage repair, specifically the repair of DNA double strand breaks (DSBs), which ensures chromosome structure maintenance. They are involved in cell cycle checkpoint activation, DNA repair via homologous recombination (HR), apoptosis, and chromatin remodeling [12,13,14]. Loss of heterozygosity (LOH) of the wild-type allele to accompany hereditary pathogenic variants in *BRCA1* or *BRCA2* is predicted to lead to loss of function (LOF) of these genes and increases genomic instability during breast cancer development [15]. A review of the BRCA Exchange database describes more than 2200 and 2600 different pathogenic variants in the *BRCA1* and *BRCA2* gene, respectively [16]; and as of June 2017, the Consortium of Investigators of Modifiers of BRCA1/2 (CIMBA) had described 1650 unique *BRCA1* and 1731 unique *BRCA2* deleterious variants [17]. These pathogenic variants are spread throughout the coding sequence of each gene, with the vast majority predicted to lead to protein truncation and loss of function. Despite their importance as the major genetic risk factors for breast cancer, pathogenic variants in *BRCA1* and *BRCA2* account for only ~20% of familial breast cancer risk [18] (Figure 1).

Thus, significant research efforts have been put into identifying other susceptibility genes for breast cancer, particularly genes that also operate in the DNA damage response (DDR) pathway. These findings are summarized in Table 1. Examples of additional breast cancer susceptibility genes identified through these studies include:

*PALB2* (Partner and localizer of BRCA2): Risks conferred by germline variants in the *PALB2* gene are comparable to the risk conferred by *BRCA2* in some familial cases. *PALB2* plays a central role in DSB repair; BRCA1 recruits PALB2 in response to DSBs, followed by recruitment of BRCA2 forming a complex on DNA. Formation of this complex is necessary for recruitment of RAD51 and consequently RAD51-mediated homologous recombination [19,20].

*ATM* (Ataxia telangiectasia mutated): Homozygous germline pathogenic variants in ATM are associated with ataxia-telangiectasia; but heterozygous variants also confer risk of breast cancer. The *ATM* gene encodes a protein kinase with a central role in DDR. Upon ATM activation by DSBs, it phosphorylates other genes involved in different stages of the DDR including *BRCA1*, *TP53*, and *CHEK2* [21,22].

*CHEK2*: After BRCA1 and BRCA2, this was the first breast cancer moderate risk gene to be identified after observing a common deletion (1100delC) in non-BRCA breast cancer families [23,24]. Functionally, CHEK2 plays a loss of function role by causing cell arrest in response to DSBs [25].

*CDH1* (E-Cadherin 1): Association of lobular breast cancer (LBC) is associated with germline pathogenic variants in CDH1, normally associated with hereditary diffuse gastric cancer (HDGC) and non-HDGC families. The product of this gene is a calcium-dependent cell adhesion protein and a key player in epithelial cell–cell interactions, affecting cell mobility and proliferation [24,26,27].

*TP53*: Germline pathogenic variants in this gene lead to the Li-Fraumeni syndrome, which is associated with the risk of developing a myriad of cancers including breast cancer. The p53 protein, commonly referred to as the guardian of the genome, is involved in a variety of DDR mechanisms via regulating cell-cycle arrest and apoptosis [28,29].

*PTEN* (phosphatase and tensin homolog): Germline risk variants in *PTEN* are observed in Cowden disease, which is characterized by a high risk of breast cancer. It is a multifactorial tumor suppressor gene with protein phosphatase activity that is involved in the PI3K/AKT-mTOR signaling pathway that controls cell cycle [30,31].

Other genes: Pathogenic variants in *BRIP1* [32], *RAD51C*, *RAD50* [33], *NBN* [34], *STK11* [35], and *RECQL* [36] may also confer some level of breast cancer risk.

The clinical importance of identifying additional breast cancer susceptibility genes is reflected in their use in multi-gene panel testing for the identification of individuals at risk of developing breast cancer [37,38]. The National Comprehensive Cancer Network^®^ (NCCN^®^) is an authority in publishing guidelines regarding the clinical management of breast cancer [39], following the identification of sequence variants in these genes. However, accurate risk estimates associated with these genes are limited, and so translating them into strategies for effective population testing and clinical management continues to be challenging. This is made more daunting because genes may also be under the influence of modifying genetic risk alleles throughout the genome and epidemiological risk factors [40], and variations in the risks of breast cancer based on the type and location of coding pathogenic variants [21,41,42,43].

Another confounding factor in accurate risk estimation may be due to the overall design of genetic studies that typically choose their patient population based on selective criteria, e.g., age at onset or limited sample size [44,45,46]. This can result in an ascertainment bias in the estimation of risk in family studies compared with the general population. Increasing the study sample size is one approach to obtain accurate risk estimates. A recent study described whole gene sequencing of 34 candidate breast cancer susceptibility genes in 113,000 subjects, most of which were from population-based studies. This study identified putative pathogenic variants in *ATM*, *BRCA1*, *BRCA2*, *CHEK2*, and *PALB2* as the most prominent genes for breast cancer, harboring protein-truncating variants that confer significant disease risks (odds ratio of 2.10 to 10.57) to overall breast cancer risk. *BARD1*, *RAD51C*, *RAD51D*, *PTEN, NF1, TP53*, and *MSH6* also confer elevated but more moderate risks (odds ratio of 1.76 to 3.06). Missense variants, although more functionally unclear, in *CHEK2*, *ATM*, *TP53*, *BRCA1*, and *BRCA2* also showed evidence of association with overall breast cancer risk (odds ratio of 1.06 to 1.42) [47]. Similarly, in a concurrently published study, Cancer Risk Estimates Related to Susceptibility (CARRIERS) reported that in addition to *BRCA1* and *BRCA2*, which were highly associated with breast cancer risk (odds ratio 7.62 and 5.23, respectively), pathogenic variants in *PALB2* and *CHEK2* were associated with a moderate risks of breast cancer (odds ratio of 3.83 and 2.47, respectively) [48]. Several genes that were previously purported to be associated with breast cancer risk did not show strong associations in these studies, notably *NBN*, *BRIP1*, *RECQL*, *FANCC*, *FANCM*, *MRE11*, *MSH2*, *RAD50*, *RINT1*, *STK11*, and *XRCC2*.

### 2.2. Common Low Penetrance Risk Alleles for Breast Cancer

Combined, pathogenic variants in the known high- and moderate-penetrance genes account for ~25% of the heritability of breast cancers (Figure 1). The missing heritability is likely due to a combination of rare and common genetic risk variants with varying levels of penetrance and prevalence in the population [49]. Common low-risk variant with minor allele frequency (MAF) of >0.05% generally confer small overall risks (less than 1.5 fold) and because of their low penetrance, cannot be identified using traditional pedigree-based linkage approaches [50]. As a result, population-based genetic association studies to identify these risk variants have gained exponential significance over the past decade and more. These studies can be categorized into three main groups based on their respective approach: 

Candidate gene-association studies: This approach focuses on the pathways or genes with a potential known biology in disease development. However, they are not agnostic and for breast cancer have largely been unsuccessful, perhaps with the exception of identifying a nonsynonymous risk associated variant in the coding region of the *CASP8* gene on chromosome 10p14 risk locus [51].

Multistage Genome Wide Association Studies (GWAS): This methodology uses a subset of samples to identify the most associated SNVs (referred to as the tag SNVs), followed by analyzing the tag SNVs in a larger set of samples [52]. The first GWAS effort by Easton et al., used this methodology and led to the discovery of 4 breast cancer risk loci within known genes. These included 10q26 (*FGFR2*), 16q12 (*TNRC9*), 5q11 (*MAP3K1*), 11p15 (*LSP1*), as well as an intergenic risk locus (8q24) [53]. Further studies conducted during the same period of time, identified additional risk variants at the 3p24, 17q22 [54], 6q25 [55], 5p12 [56], and 2q35 [57] risk loci. 

Large-scale GWAS studies and meta-analyses of multiple GWAS: In 2013, the Collaborative Oncological Gene–environment Study (COGS) used a custom array (iCOGS) and tested around 200,000 SNVs in each individual from the BCAC study. This included 45,290 and 41,880 cases and controls of European ancestry, respectively. This study not only confirmed the association between 23/27 previously identified risk loci, but also identified 41 additional novel loci associated with breast cancer risk [58]. A further 15 risk loci were subsequently identified in 2015 using the same custom array approach [59]. Over time, GWAS have increased in scale, both in terms of the number of SNVs tested and subjects analyzed in each experiment, even though the overall study design has remained the same. The most recent breast cancer GWAS used the Illumina OncoArray BeadChip platform (570,000 SNVs) to genotype 122,977 breast cancer cases as well as 105,974 control specimens of the European ancestry. This study also included 14,068 breast cancer cases as well as 13,104 control specimens of the East Asian ancestry. The authors then combined their findings with the iCOGS data as well as previous GWAS. To increase the statistical power and account for the ungenotyped SNVs that are in linkage disequilibrium (LD) with the tag SNV, genotype imputation of the 570,000 SNVs using known haplotypes (referred as a group of SNVs which get inherited together) was done (r^2^ > 0.3). The imputation analysis increased the number of SNVs under investigation to approximately 11 million. Using this large-scale study, Michailidou et al. were able to identify 65 novel breast cancer risk loci, with a significant number mapping to the non-coding DNA regions, which overlapped regulatory elements and often enriched for transcription factor binding sites (TFBSs) [60]. As a result of the comprehensive and extensive GWAS that have so far been performed, ~18% of the heritability of breast cancer has been attributed to common low penetrance risk alleles [61], Figure 1. However, the presence of thousands of additional common risk alleles awaiting identification is anticipated. A variety of crucial steps and approaches can pave the way for the much-needed identification of novel risk alleles. These include the use of larger-sized GWAS studies, accounting for ancestry-specific risk factors as well as implementation of pleiotropy effects [62].

#### Clinical Utility of Identifying Common Risk Variants for Breast Cancer

The identification of hundreds of common risk alleles for breast cancer can be used to improve the accuracy of the genetic risk prediction and population-based risk stratification. One main challenge is that the vast majority of individual common risk alleles for breast cancer are associated with very small disease risk contribution (OR < 1.5) [63]. However, it is estimated that there may be several thousand additional risk variants and the sum of their individual risks, calculated as polygenic risk scores (PRSs), can lead to significant in-risk stratification of individuals [64]. In 2015, Mavaddat et al. combined 77 breast cancer risk alleles, and using a pairwise analysis evaluated the PRS associated with this set of risk alleles (PRS77) showing that they could stratify breast cancer risk independent of family history [65]. In addition, the same group performed a PRS based on 313 confirmed risk loci (PRS313). PRS313 improved the prediction value of breast cancer risk over PRS77 (odds ratio 1.61 vs. 1.46). For overall breast cancer, a 33% lifetime risk for women in the top centile of risk was observed, which is considered high-risk based on the UK NICE definition [61]. The PRS313 model was applied for risk stratification in a recent study by the CIMBA consortium and determined a strong association between PRS and breast and ovarian cancer in individuals who carry BRCA1/2 variants. Moreover, women at both ends of the PRS spectrum were found to have a significantly different absolute risk [66]. It has also been shown that Integration of PRS313 with lifestyle, hormonal, and reproductive factors in Breast and Ovarian Analysis of Disease Incidence and Carrier Estimation Algorithm (BOADICEA) risk modeling improved disease stratification compared with common variant PRS alone [67,68,69].

Another utility of GWAS risk variants is the clinical opportunities that can arise from understanding of the novel biological mechanisms of breast cancer initiation and development, driven by germline GWAS risk variants. Innovative therapies such as poly (ADP-ribose) polymerase (PARP) inhibitor [70], or care management based on the drug response predictive value of GWAS risk variants, have also been employed as seen for immune-related disease [71]. However, the vast majority of the GWAS risk variants identified for breast cancer reside in the intergenic or intronic regions and identifying their potential target genes and candidate clinical biomarkers is challenging [72]. To overcome this obstacle, further functional studies are needed (discussed below).

### 2.3. Risk Associations with Breast Cancer Subtypes

Breast cancer is a heterogeneous disease which can be stratified into different clinical subtypes based on their biomarker expression (ER-estrogen receptor, HER2-human epidermal growth factor, and PR-progesterone receptor). The different subtypes are associated with various therapeutic regimens and prognoses, which likely reflects differences in their underlying biology. Briefly, breast cancers are mainly divided into: (1) luminal A (ER/PR+, HER2−); (2) luminal B (ER/PR+, HER2−); (3) (HER2)-enriched; and (4) basal-like triple negative breast cancer (TNBC) (ER−, PR−, HER2−), with the latter being the most aggressive form [73]. Expression of nuclear Androgen Receptor (AR) has recently emerged as another putative marker associated specifically with TNBC [74].

#### 2.3.1. Subtype Stratification for Coding Pathogenic Variants in High- and Moderate-Breast Cancer Susceptibility Genes

**Figure 2:**
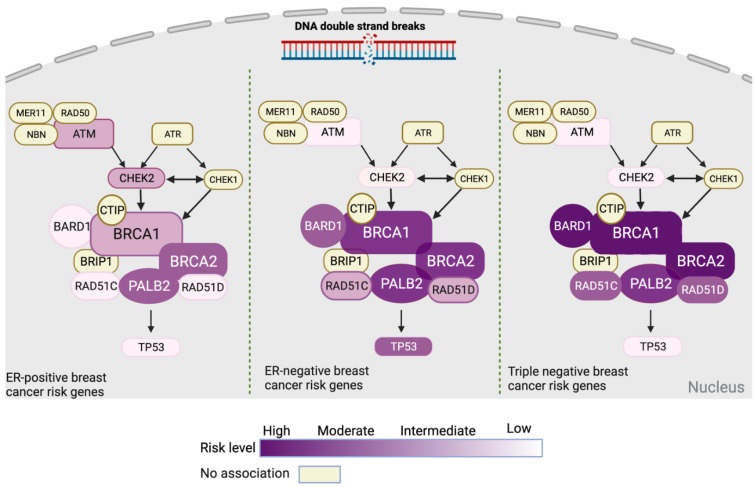
Diagrammatic representation of tumor subtype–associated breast cancer susceptibility genes present in the double-strand DNA break repair pathway based upon the recent study by Breast Cancer Association Consortium et al., 2021 [47]. ER = estrogen receptor. Figures 2–4 were created with Biorender tool (https://biorender.com/, accessed on 6 July 2021).

Targeted sequencing analyses to identify pathogenic variants in both high- and moderate-penetrance breast cancer susceptibility genes have recently provided insights into the different genes and their breast cancer subtype associations. A correlation between *BRCA1* variants and TNBC had been shown previously [44,75], whereas *TP53* germline variants may be more frequently associated with HER2-positive breast cancers than other subtypes [76,77]. The large sample size of breast cancer cases included in the recently published targeted sequencing analysis by BCAC has provided the opportunity to identify subtype-specific associations of all the cancer predisposing genes. In this study, *ATM* and *CHEK2* showed a stronger association with ER-positive breast cancer (odds ratio of 2.33 and 2.67). In the case of ER-negative breast cancer, stronger associations were observed for *BARD1*, *BRCA1*, *BRCA2*, *PALB2*, *RAD51C*, and *RAD51D*. *BARD1*, *BRCA1*, and *BRCA2* also showed a stronger association with TNBC compared with other ER-negative breast cancers [47]. Notably, missense pathogenic variants also have a subtype-specific pattern based on this study as *CHEK2* and *CDH1* are associated with ER-positive breast cancer, whereas *BRCA1* is associated with ER-negative breast cancer [47]. On the other hand, the recent CARRIERS study also reports subtype-specific risk, finding an association between *BARD1*, *RAD51C*, and *RAD51D* with increased risks of ER-negative breast cancer and TNBC, whereas pathogenic variants in *ATM*, *CDH1*, and *CHEK2* were associated with an increased risk of ER-positive breast cancer [48]. The subtype-specific risks of breast cancer based on ER status for genes in the DNA double strand break pathway are illustrated in Figure 2.

#### 2.3.2. Subtype Stratification for Risk Variants Identified by Breast Cancer GWAS

Common risk alleles identified by breast cancer GWAS also appear to confer subtype-specific risks, mainly by ER-status, suggesting the involvement of differential biological mechanisms in disease etiology. Purrington et al. performed a GWAS study on stage I TNBC cases, and identified breast cancer risk loci associated with ER-negative breast cancer [78]. An additional 15 novel breast cancer risk loci were identified in this study including *PEX14*, 2q24.1, 2q31.1, *ADAM29*, *EBF1*, *TCF7L2*, 11q13.1, 11q24.3, 12p13.1, *PTHLH*, *NTN4*, 12q24, *BRCA2*, *RAD51L1*-rs2588809, and *MKL1.* Of these, *PTHLH* reached genome-wide significance [79]. The differential association of GWAS risk variants with breast cancer subtype was also detected in the latest GWAS study where the authors reported that 21 out of 65 loci showed significant differences in their association with breast cancer based on ER status [60].

While the majority of the GWAS-identified risk variants are associated with ER-positive breast cancer, a significant proportion of them are also associated with ER-negative disease. However, a subset of these risk variants are exclusively associated with ER-negative breast cancer. In this regard, Milne et al. recently investigated ER-negative breast cancer and identified 10 novel risk variants associated specifically with the ER-negative subtype. An ER-negative association was also detected for the previously identified GWAS risk variants, bringing the total number to 125 [80].

More specifically, fine-mapping of all these breast cancer risk loci identified by GWAS found that of 196 strong risk signals, 66 (34%) had a higher risk for ER-positive breast cancer (e.g., *CCND1*, *CHEK2*, *FGFR2*), 29 (15%) with ER-negative disease (e.g., *BRCA2*, *CREBBP*, *ESR1* risk loci), while the remainder (51%) were associated with similar risks for both ER-positive and ER-negative breast cancer development [81].

PRSs for breast cancer also vary by the disease subtype. The PRS313 model was optimized to account for subtype effects and showed an improvement in predicting both ER-positive and ER-negative breast cancers [61]. A confounding factor in these predictions is that ER-negative breast cancers tend to be more aggressive and so have lower PRSs, which has also been indicated by two other studies [82,83]. Taken together, these studies suggest a shared but also somewhat different biology underlies the development of different breast cancer subtypes, which is consistent with the findings for high- and moderate-penetrance genes (Figure 2).

## 3. The Functional Consequences of Coding and Non-Coding Risk Variants for Breast Cancer

### 3.1. Interpretation of Coding Risk Variants in High- to Moderate-Penetrance Susceptibility Genes

Because of our knowledge of the genetic code, the interpretation of the functional consequence of coding variants has traditionally been more feasible than for non-coding variants, as the effects on the protein product can be predicted in silico and more readily investigated experimentally. Functional assays to study coding variants may include studying the effect of a given variant on enzymatic activity and radiosensitivity [41], the functional consequence of the variants on homologous recombination repair and cell viability [84], and mouse embryonic stem cell (mESC)-based functional assays [85].

For a more comprehensive assessment and standardized interpretation of the pathogenicity of sequence variants, The American College of Medical Genetics and Genomics (ACMG)/Association for Molecular Pathology (AMP) has developed practice guidelines [86]. ACMG/AMP recommends a five-tier evidence-based classification system for genetic alterations including population, computational and predictive, allelic, segregation, inheritance, and functional data. Functional evidence is considered as strong evidence in this regard. According to the ACMG/AMP criteria, sequence variants can be classified as pathogenic, contributing to the development of disease; likely pathogenic, with a greater than 90% certainty that a variant contributes to disease development; variants of unknown significance (VUS), not classified yet and awaiting further information; likely benign, without a major effect on tumorigenesis; and benign, which are not disease-causing [86]. The ClinGen’s Variant Curation Expert Panels (VCEPs) also use the ACMG/AMP guidelines and have customized them for genes such as *TP53* [87], *PTEN* [88], and *CDH1* [89]. This customized approach has resulted in a reduction in the number of VUSs in these genes.

Frameshift indels and nonsense variants are categorized as deleterious, because they introduce premature termination codons—for example the p.Tyr1853* (* represents a stop codon) variant in *BRCA1* [90]. While the presence of protein-truncating variants is often presumed pathogenic, this is not always the case. For example, a recent study using an mESC-based functional assay investigated the effects of variants of exon 12 of BRCA2 on its function [91]. They demonstrated that several nonsense variants in this exon affect the splicing site and lead to the exon skipping while partially maintaining protein function. In another example, Mazoyer et al., in 1996 reported the identification of the germline nonsense substitution of BRCA2 c.9976A > T; p.Lys3326*, that leads to the generation of a stop codon in BRCA2 and loss of the last 93 amino acids. Yet, no evidence of increased susceptibility to breast and ovarian cancers was observed, suggesting that the C-terminal may not be necessary for the tumor suppressor activity of BRCA2 [92]. In a large-scale breast cancer GWAS study in 2013, the BRCA2 c.9976A > T variant was shown to be a low penetrance risk allele for breast cancer. Meeks et al. performed an expanded analysis of this association with breast cancer risk and reported an independent risk association with this variant in both breast and ovarian cancer cases [58,93].

There still remain challenges in establishing the function of VUSs, which may be of clinical significance given they occur in highly penetrant susceptibility genes, and if established as truly pathogenic, would provide opportunities for clinical interventions such as risk-reducing surgery. In this regard, as of June 2021 there were 3083 and 5483 VUSs for *BRCA1* and *BRCA2*, respectively, reported in the ClinVar database [94]. An example of a VUS demonstrated to have a pathogenic effect is the synonymous c.516G > A (p.Lys172=) variant in exon 6 of *BRCA2*, which leads to skipping of exons 5 and 6, due to the generation of a splicing site. Based on the functional results of this study, this variant was included in genetic testing [95]. However, this is one variant of several hundred candidate variants in these genes and the detailed, robust analysis of the coding variant function remains a bottleneck that needs to be addressed through consortia/community approaches and/or high-throughput functional screening assays as they evolve.

In an effort to circumvent this issue, Findlay et al. used high-throughput saturation genome editing (SGE), a CRISPR/Cas9-based approach, to study the functional effect of potential SNVs (~4000) in the genomic regions encoding two functional domains of *BRCA1* known as the RING (exon2–5) and BRCT (exon15–23). This study used a functional scoring system to measure the deleterious effect of SNVs over time in a haploid cell line (HAP1), representative of the functional effect of the SNV on BRCA1 function. In this system, synonymous SNVs generate a score of 0, and variants with LOF effects produce a negative score (i.e., −2.12 score for nonsense variants). They were able to categorize SNVs into three main functional classes. Their results indicated that 72.5% of the variants were functional, 21.1% were non-functional, while 6.4% had intermediate function [96]. This observation is in consistency with previously established assessments of pathogenicity, suggesting that incorporation of functional-based evidence would improve the power of VUS classification in high-penetrance genes.

### 3.2. Functional Characterization of Common Low-Penetrance-Risk Alleles

The challenges are different for common low-risk alleles identified by GWAS. Although a large number of risk variants have been identified for breast cancer [60,80], more than 90% of these lie in non-protein coding DNA [97,98] for which there is no robust genetic code to interpret their function. In addition, GWAS risk alleles are associated with small individual risks which implies the functional impact of each variant is also likely to be small. Delineating the biological relevance of non-coding risk variants is complex as shown by the few studies that have focused on functionally validating these risk alleles (e.g., the published works on 10q26 [99], 11q13 [100], 2q35 [101], and 19p13 [102]). Non-coding risk variants likely impact the activity of regulatory elements (REs) and TFBSs, which in turn affects the expression of a target susceptibility gene and/or gene expression networks. Given the complex nature of transcriptional regulation, which is tissue specific, the challenges in interpreting these non-coding variants are: (i) to identify the credible causal risk variant(s) associated with disease development; (ii) to identify the susceptibility gene(s) at risk loci and their functional role in disease pathogenesis; and (iii) to establish the regulatory mechanisms by which risk variant(s) affect the expression of their target gene(s). The field of functional genomics (FG) plays a critical role in overcoming the ever-increasing bottleneck between discovery and function with the ultimate goal to go from risk variants to the target gene, and mechanistic insights. FG does this by using a variety of complementary multi-omics datasets and approaches including bioinformatics, transcriptomics, and proteomics to identify the causal variants and genes, and it recruits molecular and cell biology assays to validate the causality and identify molecular pathways [103].

#### 3.2.1. Identifying the Credible Causal Risk Variant and the Target Regulatory Element

GWAS studies are usually based on genotype data from SNP-arrays and generally only identify risk variants that tag multiple other risk-associated variants in the region due to linkage disequilibrium. While a small number of variants at a locus may represent the most statistically significant risk allele(s) in reality, other statistically weaker variants in LD may be the causal disease drivers [104]. Thus, the first step in identifying the causal allele often requires obtaining a comprehensive catalog of all genetic variants correlated with the primary association signal at each risk locus. Large consortia-based genotyping efforts, combined with imputation methods that utilize the vast catalog of genetic data in the 100,000 Genomes Project can ‘fine-map’ the genetic content of a risk locus to identify the most likely causal variants. For example, BCAC and CIMBA have recently fine-mapped ~150 breast cancer risk regions [81] identifying 352 independent risk signals and >13,000 candidate causal risk variants. Using these signals improved the proportion of heritability for breast cancer due to common variant risk alleles by 6% compared with using only the GWAS-identified tag SNVs.

Non-coding risk variants may reside in the REs in a risk region including gene promoters, enhancers, silencers, and insulators, where they perturb TFBSs or local chromatin structure, ultimately resulting in changes of transcriptional output of one or more target gene(s) [60,81,105]. Catalogs of genome-wide REs for many human tissues have been generated and are available publicly through initiatives such as ENCODE (ENCyclopedia Of DNA Elements) [106], the Epigenomics Roadmap [107], and FANTOM [108]. At least a proportion of epigenomic features are likely to be tissue-/disease-specific and can be identified through enrichment of risk alleles with REs. The most recent breast cancer GWAS identified a 2–5 fold increased enrichment of risk SNVs in REs, mostly mapping to the binding sites for breast cancer–associated transcription factors including FOXA1, ESR1, GATA3, E2F1, and TCF7L2 [60]. The latest breast cancer fine-mapping analyses also combined GWAS data with breast-specific genomic features using publicly available data for seven normal breast cell lines and 19 breast cancer cell lines. This analysis identified enrichment for CCVs in open chromatin, actively transcribed genes, gene regulatory regions as well as TFBSs. ESR1, FOXA1, GATA3, and EP300 TFBSs were enriched in CCVs for overall breast cancer. However, the enrichment for ESR1, FOXA1, and GATA3 was stronger for ER-positive CCVs than for ER-negative or overall breast cancer CCVs. A subtype-specific enrichment was also observed for ER-positive breast cancer cell lines with a significantly larger overlap of CCVs with REs, compared with ER-negative cell lines [81]. Intriguingly, the enrichment of CCVs in TFBS is consistent with the hypothesis that they regulate the expression of their target genes by altering the TFBS within the REs. The 19p13.11 and 19q13.31 breast cancer risk loci demonstrate this, where the risk variants modify the TFBS and affect the expression of *ZNF404* and *ANKLE1* genes in breast tissue [109].

#### 3.2.2. Identifying the Target Susceptibility Gene(s) at Risk Loci

Relationships between inherited variation, expression, and trait can be investigated through approaches such as local expression quantitative trait locus (eQTL) analysis [110], PrediXcan [111], and transcriptome-wide association scans (TWAS) [112]. These approaches use data from large datasets such as Genotype-Tissue Expression (GTEx) and The Cancer Genome Atlas (TCGA), which profile and genotype the transcriptomes of normal and cancer tissues and then integrate the two to identify associations between risk allele and gene expression at the transcript level to identify candidate susceptibility genes and the downstream pathways affected by risk alleles. As with epigenetic regulation, gene expression is tissue specific and so eQTL analyses are most informative when performed using samples specific to the disease under study. However, the use of eQTL from tissues other than the breast (e.g., adipose tissue) has also been used to increase statistical power to identify potential target genes [113]. The recent popularity in the use of single-cell eQTL may also lead to the identification of single-cell transcriptomes and shed light on the effect of risk variants across cell-subtypes inside a tissue [114]. Examples of putative breast cancer susceptibility genes identified using eQTL-based approaches include: *ABHD8* at 19p13 [102], *AKAP12/ESR1* at 6q25.1 [115], and *MYC* at 8q24 [116]. Fachel et al. also reported a 60% overlap between the CCVs and sequence variants affecting gene expression [81]. More specifically, a study by Fagny et al. used cis- and trans-eQTL network analysis to understand the biological mechanisms underlying GWAS risk variants in different cancers including breast, and found risk-associated SNVs that likely control the expression of oncogenes, tumor suppressor genes, and immune function genes [117].

#### 3.2.3. Identifying Susceptibility Gene-Regulatory Interactions at Risk Loci

Common variants that influence the activity of specific REs such as enhancers may affect target gene expression through a direct, physical interaction, acting in *cis* or *trans* [118,119]. Therefore, studying the effect of variants in the context of the 3D chromatin structure enables the identification of potential target genes associated with REs and risk variants. The 3D genome can be studied using chromosome conformation capture (3C)-based techniques, which identify regions of physical contact between gene promoters and REs [120]. Where eQTL analyses have identified a candidate gene, the gene promoter can be used to anchor the assay (i.e., circularized chromosome conformation capture or 4C) to identify the likely causal SNVs which will also confirm the target gene [121]. In the absence of an identifiable eQTL association, the region(s) spanning causal risk SNVs can also be used to identify a likely gene target as shown at the 8q24 risk locus for breast, ovarian, prostate, and colon cancers [122]. At a genome-wide scale when fine-mapping has identified several candidate SNVs, high-throughput, genome-wide approaches, such as Hi-C [123] and HiChiP [124], can be used to study the genome-wide interactome maps and identify the interaction of REs with risk regions identified by GWAS. Hi-C has been used to investigate the role of RUNX1 [125], ER [126], and GATA3 [127] on chromosome conformation in tumorigenesis and development of breast cancer.

Fachel et al. used a prioritization approach for potential target gene identification, using multiple lines of evidence as discussed above including eQTL, functional annotation, and interaction-based data. In total, the authors were able to prioritize 191 potential target genes for 150 breast cancer risk regions, mostly breast cancer associated transcription factors and somatic driver genes including *CREBBP*, *EP300*, *ESR1*, *GATA3*, and *MYC*. Pathway analysis in ER-positive GWAS identified potential target genes with biological roles in mammary development (23%), immune system pathways (18%), and nuclear receptor pathways (17%). Interestingly, 19% of the ER-negative target genes belonged to the DNA integrity checkpoint processes and the immune system, and 16% were involved in apoptotic processes. These data also suggest underlying mechanistic differences or differential pathways specific to each breast cancer subtype [81].

#### 3.2.4. Experimental Approaches to Validate the Functional Effect of Causal Variants and Target Genes

**Figure 3:**
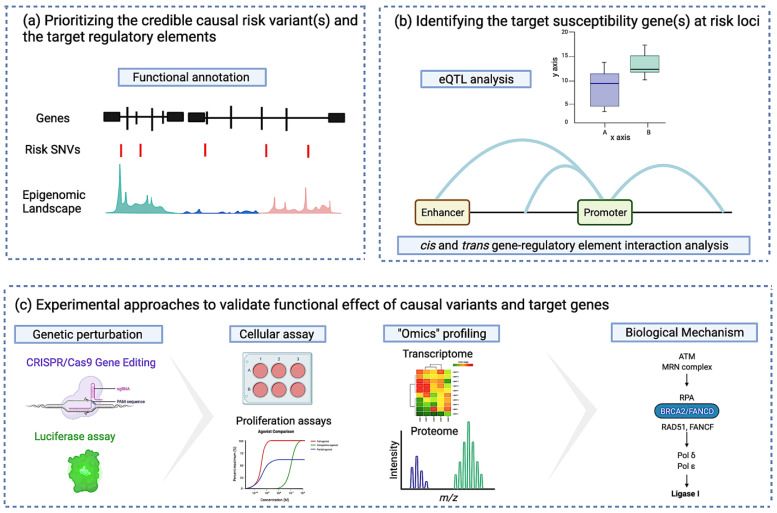
Illustration of functional follow-up analysis of non-coding low penetrance common risk variants. (**a**) Integration of genotype and epigenomic data to identify the candidate regulatory targets of risk variants; (**b**) Integrative analysis of the correlation between genotype to gene expression paired with gene-regulatroy element interaction, identifies putative candidate susceptibility genes; and (**c**) genome editing, multiomics, and cell biology assays are utilized to functionally evaluate noncoding DNA elements and their potential target genes to uncover their role in neoplastic development.

Although using annotation and predictive tools will help to prioritize the list of the risk variants and target genes, the most likely causal risk variants and target genes need to be validated experimentally. Genome editing (GE) technologies can be used to assess the consequence of risk SNVs that lie in the non-coding genome, and to introduce protein coding changes that abrogate the function of potential target genes followed by testing the effect of gene editing on neoplastic development [128]. Various customizable nucleases have been described, with the clustered regularly interspaced short palindromic repeat (CRISPR)-associated (Cas9) the most widely adopted. Cas9 nuclease can be directed to cleave a site of interest by a short guide RNA (gRNA) with complementarity to the target DNA sequence, if this sequence is proximal to a specific protospacer adjacent motif (PAM) recognized by Cas9 (e.g., Cas9 requires a PAM of the form 5′-NGG) [129]. CRISPR screens (knock-out, activation (CRISPRa), and interference (CRISPRi)) to perturb variants/genes that will modify the expression of candidate genes in experimental models of breast, followed by whole transcriptome analysis using RNA-seq will identify differentially expressed genes (DEGs) and networks associated with breast cancer risk loci [130]. After using the whole transcriptome as a read-out to further narrow down causal variants and genes, their effects on neoplastic phenotypes can be studied using cell biology assays. Another variant editing tool that can confirm the role of risk REs on gene expression is massively parallel reporter assays (MPRAs) [131]. This approach has been used to study the mechanisms underlying GWAS identified in immune-mediated disease [132]. STARR-seq is an example of an MPRA assay that has gained attention and has shown potential for high-throughput analysis of multiple risk variants [133,134]. Figure 3 summarizes a step-by-step functional framework to establish the functional mechanisms underlying GWAS risk loci as discussed in this review.

## 4. Conclusions

**Figure 4:**
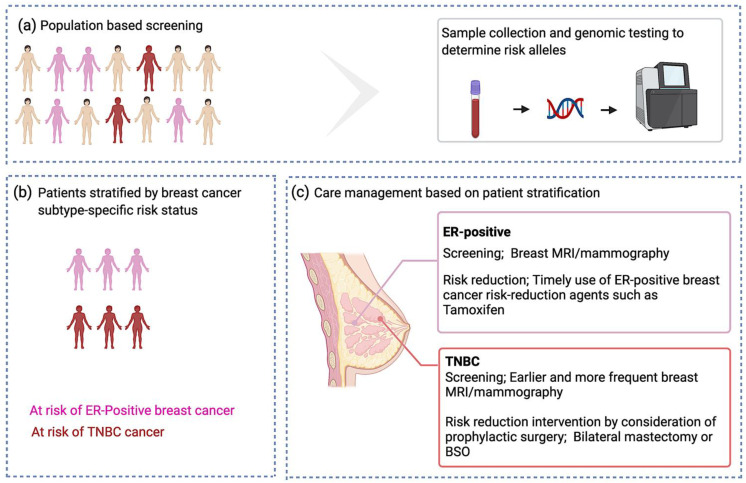
Breast cancer subtype-specific screening and preventive approaches proposal. (**a**) Genetic testing to identify risk alleles; (**b**) stratification of at-risk individuals based on the presence of breast cancer subtype-specific risk alleles; and (**c**) Subtype-specific care management.

A timely and refined breast cancer risk assessment on a population scale is challenging and relies on accurate risk estimates based on the individualized genetic profiles. An important step to achieve this seems to be subtype-specific risk stratification of individuals undergoing molecular genetic testing. Risk stratification will not only improve the accuracy of early detection and screening, but also allow targeted risk management and clinical care programs. Adding subtype-specificity in risk discrimination could benefit those people in need of screening and/or surveillance, Figure 4. For example, for those at risk of developing TNBC, earlier and more frequent mammography, and breast magnetic resonance imaging (MRI) to identify the pre-malignant disease could be an option for early detection and prevention. Individuals at risk of disease might benefit from prophylactic mastectomy to reduce their risk [135]. In addition, the use of risk-reduction agents such as selective estrogen receptor modulators (SERMs) may provide opportunities for subtype-specific prevention and therapy. For example, tamoxifen and raloxifene may work as primary prevention agents for ER-positive breast cancer [136,137]. Trastuzumab and lapatinib are anti HER-2 targeted therapies and may work for prevention of HER2+ breast carcinomas; bicalutamide and enzalutamide are AR antagonists that may impact the disease course of AR+ TNBCs [138]. The difference in subtype-specific genetic profiles as seen for both high- and moderate-penetrance genes, and for PRS313 (sum of common low penetrance alleles), could indicate different mechanisms underlying these cancers. Understanding the functional mechanisms of subtype-specific risk alleles using FG approaches may also lead to the identification of subtype-specific novel biomarkers for screening and treatment. This paradigm is best exemplified in *BRCA1* and *BRCA2* pathogenic variant carriers where knowledge of the function of these genes led to the development of poly (ADP-ribose) polymerase (PARP) inhibition as a novel therapy for the treatment of breast cancers [70]. We envision that with the help of FG tools discussed herein, additional diagnostic and therapeutic avenues could be explored and harnessed for a substantial portion of patients without established genetic heritability that carry sequence variants in the non-coding genome.

## Figures and Tables

**Table 1:** Summary of high- and moderate-penetrance breast cancer susceptibility genes.

Gene	Genomic Location	Odds Ratio * (95% CI)	Associated with Other Cancers #	Syndrome	Absolute Risk #
*ATM*	11q22	2.1 (1.71–2.57)	Ovarian, Pancreatic	Ataxia-telangiectasia	15–40%
*BARD1*	2q35	2.09 (1.35–3.23)			Insufficient data to define
*BRCA1*	17q21	10.57 (8.02–13.93)	Ovarian, Pancreatic, Prostate		>60%
*BRCA2*	13q12	5.85 (4.85–7.06)	Ovarian, Pancreatic, Prostate, Melanoma		>60%
*CHEK2*	22q12	2.54 (2.21–2.91)	Colon		15–40%
*PALB2*	16p12	5.02	Ovarian, Pancreatic	Fanconi anemia	41–60%
*PTEN*	10q23	2.25 (0.85–6.00)	Thyroid, Colon, Endometrial	Cowden’s syndrome	40–60%
*RAD51C*	17q23	1.93 (1.20–3.11)	Ovarian		15–40%
*RAD51D*	17q12	1.8 (1.11–2.93)	Ovarian		15–40%
*TP53*	17p13	3.06 (0.63–14.91)	Pancreatic, and cancers associated with Li-Fraumeni syndrome	Li-Fraumeni syndrome (expanded to heritable TP53-related cancer (hTP53rc) syndrome)	>60%

* Overall breast cancer risk associated with protein truncating variants based on the most recent breast cancer association study. # Based on the most recent The National Comprehensive Cancer Network^®^ (NCCN^®^) Clinical Practice Guidelines in Oncology.
